# T3DB: the toxic exposome database

**DOI:** 10.1093/nar/gku1004

**Published:** 2014-11-05

**Authors:** David Wishart, David Arndt, Allison Pon, Tanvir Sajed, An Chi Guo, Yannick Djoumbou, Craig Knox, Michael Wilson, Yongjie Liang, Jason Grant, Yifeng Liu, Seyed Ali Goldansaz, Stephen M. Rappaport

**Affiliations:** 1 Department of Computing Science, University of Alberta, Edmonton, AB T6G 2E8, Canada; 2 Department of Biological Sciences, University of Alberta, Edmonton, AB T6G 2E9, Canada; 3 National Institute for Nanotechnology, 11421 Saskatchewan Drive, Edmonton, AB T6G 2M9, Canada; 4 Center for Exposure Biology, School of Public Health, University of California, Berkeley, CA 94720-7360, USA

## Abstract

The exposome is defined as the totality of all human environmental exposures from conception to death. It is often regarded as the complement to the genome, with the interaction between the exposome and the genome ultimately determining one's phenotype. The ‘toxic exposome’ is the complete collection of chronically or acutely toxic compounds to which humans can be exposed. Considerable interest in defining the toxic exposome has been spurred on by the realization that most human injuries, deaths and diseases are directly or indirectly caused by toxic substances found in the air, water, food, home or workplace. The Toxin-Toxin-Target Database (T3DB - www.t3db.ca) is a resource that was specifically designed to capture information about the toxic exposome. Originally released in 2010, the first version of T3DB contained data on nearly 2900 common toxic substances along with detailed information on their chemical properties, descriptions, targets, toxic effects, toxicity thresholds, sequences (for both targets and toxins), mechanisms and references. To more closely align itself with the needs of epidemiologists, toxicologists and exposome scientists, the latest release of T3DB has been substantially upgraded to include many more compounds (>3600), targets (>2000) and gene expression datasets (>15 000 genes). It now includes extensive data on ‘normal’ toxic compound concentrations in human biofluids as well as detailed chemical taxonomies, informative chemical ontologies and a large number of referential NMR, MS/MS and GC-MS spectra. This manuscript describes the most recent update to the T3DB, which was previously featured in the 2010 NAR Database Issue.

## INTRODUCTION

The modern world is full of toxic substances. Some are relatively benign and only lead to disease or injury after years of exposure, others are acutely toxic and can lead to death within seconds of contact or ingestion. The economic and social impact of our exposure to toxic substances is often little known and seriously underestimated. For instance, acute poisoning is now the leading cause of ‘non-disease’ death and injury in North America. More than 43 000 deaths/year and more than 1.1 million injuries/year have been attributed to the deliberate or accidental overdose of toxic substances (1,2). Adverse drug reactions (ADRs), which are essentially non-acute or sub-acute poisonings, cause between 75 000–137 000 deaths/year, 1.7–2.7 million serious events/year and are associated with a cost-of-illness that is estimated to be >$170 billlion/year in the US alone (3,4). Indeed, ADRs are thought to be the fourth and seventh leading causes of death in the US and Europe (4,5). Chronic exposures to certain chemicals can also have a profound impact on human health. Exposure to radon, ozone, lead, workplace chemicals, cigarette smoke, wood smoke (from cooking fires) and air pollution account for 27% of global chronic disease mortality (6). Dietary exposures leading to high plasma levels of cholesterol, alcohol, sodium and glucose account for another 23% of chronic disease deaths (6). Recent evidence indicates that endogenous uremic toxins (7,8), atherotoxins (9) and oncometabolites (10) arising from natural metabolism or food-gut microflora interactions appear to account for a significant portion of kidney disease, cardiovascular disease, diabetes, dementia and cancer (which are among the leading causes of death and disease in the developed world).

Based on the above data it appears that what chemicals humans are exposed to, either through their diet, home, workplace or environment, has a profound effect on their health. Indeed, the latest statistics indicate that toxic compound exposure (i.e. the ‘toxic exposome’) is probably the leading cause of human morbidity and mortality in both the developing and developed world (6,11). Identifying which compounds are toxic (acutely or chronically), at what levels and how they cause injury, disease or death is a challenging task. Indeed, this process has required decades of work and the coordinated efforts of many agencies (IARC, WHO, FDA, US EPA, ECHA) employing many thousands of skilled specialists including analytical chemists, environmental chemists, disease specialists, toxicologists, pharmacologists and epidemiologists. The diversity of disciplines and the long periods of time required to validate key findings means that much of the critical information about toxic compounds or the ‘toxic exposome’ is scattered through thousands of different journals, books, white papers, private holdings and on-line databases.

In an effort to consolidate this highly dispersed, multi-disciplinary data about the toxic exposome and to make it freely accessible to a broad community of researchers, we developed the Toxin-Toxin-Target Database (T3DB). Originally published in 2010 (12), the first version of T3DB contained data on ∼2900 common toxic substances along with detailed information on their chemical properties, descriptions, targets, toxic effects, toxicity thresholds, sequences (for both targets and toxins), mechanisms and references. To more closely align itself with the current needs of exposome researchers and other ‘omics’ scientists, the latest release of T3DB has been substantially upgraded to include many more compounds, targets and gene expression datasets along with extensive toxic compound concentration or exposure data. Other information has also been added including detailed chemical taxonomies, informative chemical ontologies and a large number of referential nuclear magnetic resonance (NMR), tandem mass spectrometry (MS/MS) and gas-chromatography mass spectrometry (GC-MS) spectra. Additionally, much of the previously existing data in T3DB has been rewritten, corrected or enhanced, and the web interface and database searching functions have been significantly improved. A more detailed description of all of the improvements and additions to T3DB is provided in the following pages.

### DATABASE UPDATES AND ENHANCEMENTS—OVERVIEW

The latest version of T3DB is modeled very closely after the original version in terms of general content, layout and intention. As before, T3DB's central objective is to provide in-depth, quantitative and molecular-scale information about toxic substances (i.e. the toxic exposome) and their associated targets. This molecular interaction information is further supplemented with a number of database fields covering general descriptions, structural data, nomenclature, physico-chemical data, external links, detailed descriptions of the mechanism of toxicity, metabolism, lethal or toxic dose levels, potential carcinogenicity, exposure sources, symptoms or health effects, suggested treatment options, references and targets. In addition to these fields, a number of other data fields have been added to the latest version of T3DB. Table 1 provides an overview of all of the changes incorporated into the newest version of T3DB along with a detailed comparison between version 1.0 (the 2010 version) and the current version (version 2.0). As can be seen in this table we have increased the number of data fields in T3DB from 80 to 97, the number of toxic compounds by more than 25% and the number of toxin targets by 72%. We have also introduced new toxin-induced gene expression data for more than 15000 genes. All 3679 compounds in T3DB now have either a structure or a sequence and all compounds have been classified into a formal hierarchical chemical taxonomy. Eight hundred and eighty-nine compounds have ‘normal’ or typical concentration data while 657compounds have abnormal or toxic concentration data. Binding constants and/or AC_50_ values for 1003 toxins that interact with 594 target proteins are now available. All 3679 compounds have been assigned a ‘Chemical Type’ description while another 887 compounds have ChEBI ontologies describing their specific chemical roles, biological roles and applications. In addition, MSDS (Material Safety Data Sheets) for 2410 compounds have been uploaded to T3DB. To assist with toxic compound identification or quantification by analytical chemists, more than 5100 NMR and MS spectra of pure compounds have been added to T3DB along with new tools to view and search the spectra. Additionally many changes and improvements have been made to the existing data in the database.

**Table 1.** Comparison between the coverage in T3DB version 1.0 and version 2.0

Category	1.0	2.0
No. of compounds	2896	3679
No. of unique targets	1215	2084
No. of upregulated/downregulated genes^a^	0	15 842
No. of data fields per ToxCard	80	97
No. of search types	4	9
No. of physchem parameters per compound	8	23
No. of compounds with chemical taxonomic data^a^	0	3679
No. of compounds with ontological data^a^	0	887
No. of synonyms	32 276	42 422
No. of references	2937	18 058
No. of external database links	12	15
No. of pathways^a^	0	384
Percentage of compounds with descriptions	99.6%	100.0%
Percentage of compounds with structures	93.4%	100.0%
Percentage of compounds with MSDS data sheets^a^	0.0%	65.5%
Percentage of compounds with targets	90.5%	95.8%
No. of compounds with mechanism of toxicity	2895	3256
No. of compounds with LD50 data	393	1,170
No. of compounds with normal concentrations^a^	0	889
No. of compounds with metabolism data	2494	2907
No. of compounds with symptoms	2714	2868
No. of compounds with treatment	1761	2337
No. of compounds with target binding constant data^a^	0	1003
No. of compounds with NMR spectra^a^	0	746
No. of compounds with MS spectra^a^	0	1170

^a^New data.

To describe the latest changes and enhancements in more detail, we have chosen to group them into six broad categories covering: (i) new toxins and toxic substances; (ii) new targets; (iii) chemical ontologies and taxonomies; (iv) spectral data; (v) database remediation and minor additions and (vi) interface improvements.

### New toxins and toxic substances

The original version of T3DB attempted to capture information about common poisons, toxins and toxic substances such as pollutants, pesticides, preservatives, drugs, cosmetic toxins, dyes and cleaning compounds. Many of these compounds are xenobiotics and most would be classified as acutely toxic. For this year's release of T3DB and in an effort to better capture the toxic exposome we decided to gather more information on relatively benign, naturally occurring or chronically toxic compounds. These include chemicals such as 2-hydroxyglutarate (an oncometabolite), trimethylamine-N-oxide (a uremic toxin and an atherotoxin), glucose (associated with diabetes), fructose (associated with obesity and gout) and cholesterol (associated with hyperlipidemia and heart disease). Acute doses of these chemicals are not terribly toxic, but over several decades, higher than normal plasma concentrations are known to lead to chronic diseases (6,11). A number of other endogenous metabolites (such as amino acids and organic acids) were also added from the Human Metabolome Database (HMDB) (13) due to their known neurotoxicity or developmental toxicity for individuals with inborn errors of metabolism (such as phenylalanine in phenylketonuria and homogentisic acid in alkaptonuria). In addition to these endogenous compounds an additional set of 49 known or suspected carcinogens from IARC (the International Agency for Research on Cancer), a set of more than 175 new drugs from DrugBank (14), a collection of more than 300 common pesticides, cosmetic agents, drugs and workplace chemicals from ToxCast (15), and a compilation of nearly 60 fungal toxins, phytotoxins and marine biotoxins from the Kyoto Encyclopedia of Genes and Genomes (KEGG) (16) were added to the database. In total 783 toxic compounds with chronic or acute toxic effects were added to T3DB to provide more complete coverage and a more holistic view of the toxic exposome. Currently there are 3543 small molecule toxins (<1500 Da) and 136 peptide or protein toxins (>1500 Da) in T3DB. This corresponds to an increase of nearly 800 small molecule toxins in version 2.0 over version 1.0.

While toxicity levels and lethal dose data (both of which are in T3DB) are important for understanding acute toxicity, many researchers in molecular epidemiology, metabolomics and exposome science are interested in levels of chronic exposure or healthy reference ranges—especially for endogenous or chronically toxic compounds. To address this issue we have now added normal and abnormal human biofluid concentration data for a total of 933 compounds in T3DB. The biofluids include blood, urine, saliva and cerebrospinal fluid. Each concentration entry includes the biofluid type, an average concentration value, a range (or standard deviation), gender information, a subject age range and a reference. This information was obtained from the literature, the HMDB (13), the latest NHANES study (17) and calculated from DrugBank (14).

### New targets

A key strength of T3DB has been its extensive collection of toxin or poison targets. Not only does T3DB provide the most completely referenced collection of toxin targets, it also provides detailed descriptions of how the toxins interact with their targets through the ‘Mechanism of Toxicity’ data field. As with the previous version of T3DB, extensive literature reviews were conducted to expand or correct the past list of toxin targets, to update the mechanisms of toxicity and to add additional information about each target molecule (DNA, protein or small molecule). In total, 869 new and unique toxin targets were added to version 2.0 of T3DB. Altogether 96% of the toxic compounds in T3DB now have at least one toxin target. In addition to the manual literature updates, a substantial amount of new and very useful target information was mined from the latest ToxCast studies (15). ToxCast is a multi-year, multi-million dollar effort by the US EPA to measure *in vitro* activities of nearly 2000 toxic compounds against a large panel of enzymes, receptors and cell types from multiple organisms. Using the available or published ToxCast data we selected only those target proteins that were human proteins and limited the set to ToxCast toxins that exhibited AC_50_ values of <10 μM. AC_50_ is the concentration at which the assay is activated or inhibited by 50% when compared to the control values. The value of 10 μM was chosen because it is unusual to find xenobiotic or toxic compounds at concentrations above this level in human blood or tissues. The inclusion of the ToxCast data led to the addition of up to 32 targets for some toxic compounds and an expansion of the total target list in T3DB by some 226 different proteins. While the ToxCast consortium has measured data on 2000 compounds, only 302 compounds were added to T3DB because a large number of ToxCast compounds were either failed experimental drugs or experimental compounds that will likely never be seen outside specialized labs. In addition to the AC_50_ data, protein-toxin binding constant information (*K*_d_ and *K*_i_,) was also obtained from literature surveys, DrugBank and BindingDB (18) for an additional 510 protein targets. We believe the addition of quantitative binding and activation data to T3DB should be particularly useful for developing quantitative structure activity relationships that could be used to predict toxicity, potential targets and mechanisms of action of newly identified toxic compounds.

While many toxic compounds bind to a few specific macromolecular targets with high affinity, they also can lead to substantial changes in gene expression for large numbers of genes. Most gene expression changes are largely downstream of a target-binding event, but they can have profound and long lasting biological effects. Gene expression changes can also give useful insights about the etiology or mechanisms of action for specific toxic compounds. Given their utility in modern toxicology and toxicogenomic studies, we decided to add this information to the latest version of T3DB. To obtain gene expression data we extracted specific information about the up- and down-regulated genes from previously published microarray and RNAseq experiments that are contained in the Comparative Toxicogenomics Database (19). In-house software and further manual curation was then used to generate additional gene annotation (gene name, chromosomal locations, sequence, etc.) for each of the extracted genes. In total our curation team added toxin-induced gene expression data for more than 15 000 genes with 2227 references corresponding to 415 toxic compounds.

### Chemical ontologies and taxonomies

Chemical compounds such as drugs, organic acids and lipids have long been classified into various categories based on their structures, substituents and/or chemical composition. These kinds of chemical classifications or chemical taxonomies are particularly useful for rapidly searching, comparing, extracting or clustering similar chemical entities. They can also be used to predict a variety of chemical and/or biological properties. As part of our ongoing effort to consistently classify compounds in HMDB and DrugBank (13,14) we developed a fully automated, self-consistent, structure-based chemical classification tool called ‘ClassyFire’. ClassyFire classifies compounds into a hierarchical framework consisting of ‘Kingdoms’, ‘Superclasses’, ‘Classes’, ‘Subclasses’, ‘Direct Parents’, ‘Alternative Parents’, ‘Molecular Framework’, ‘Substituents’ and ‘External Descriptors’ using well-defined rules based on their structural features and structure similarities. We applied ClassyFire to all 3679 compounds in the T3DB and validated the taxonomic assignments through manual spot checks. The ClassyFire taxonomy was used to help group the complete collection of T3DB compounds into 15 broad categories that are viewable through T3DB's ‘Class Browse’ tool.

Every compound in T3DB has a free-form, curator-composed compound description. These descriptions can range from as few as 20 words to as many as 500 words. However, chemical entities can also be described more succinctly using defined ontologies and structured vocabularies. For instance, T3DB has its own limited ontology data field (called Chemical Type) that uses one and two-word phrases to describe compounds in terms of their chemical structures and/or functions. Chemical Type information is provided for 3679 compounds in T3DB. Another ontology descriptor in T3DB called ‘Uses/Applications’ which provides a free text description of the compound's known uses, origins, industrial, medical or home applications. This data field is filled in for 3017 compounds. To further supplement T3DB's chemical ontology we have added ChEBI (20) ontology terms for another 887 compounds. The ChEBI ‘role’ ontology is particularly attractive because it OBO (Open Biomedical Ontologies) compliant and uses a defined vocabulary to describe chemical roles, biological roles and applications (industrial or otherwise). Over the coming year we intend to generate ChEBI ontological descriptions for all 3679 compounds in T3DB.

### Spectral data

As the fields of toxicology, exposomics and metabolomics continue to evolve, we believe there is an increasing need to develop databases that are compatible with their growing analytical chemistry needs. In particular, referential MS and NMR spectra of pure compounds are particularly important for identifying and/or quantifying compounds in biological matrices. To meet these compound identification/characterization needs, 1660 NMR and 3444 MS reference spectra for more than 1200 different compounds have been added to T3DB. Many of these spectra were experimentally acquired by our laboratory (13,14) while others have been assembled from freely available on-line sources (21,22). In addition to including these reference MS and NMR spectra, we have also added a number of advanced spectral viewing and spectral/mass matching tools (originally developed for the HMDB (13)) to facilitate experimental metabolomic and exposomic studies. Using these tools, users can submit chemical shift or m/z lists and search against T3DB's spectral library for exact or even approximate matches. Users can also browse, zoom and view all of T3DB's spectral data to visually compare results and assess their spectral matches.

### Database remediation and minor additions

Data currency and data quality have always been top priorities for the T3DB curation team. As a result, significant efforts over the past year have been directed at improving the quality of existing ToxCards and keeping them as current as possible. Because new compounds are constantly being added to ‘toxic watch lists’ and existing toxic compounds are constantly being further characterized or analyzed, our curation team has been actively combing the literature for information to update existing ToxCards or to add new ToxCards. As a result, hundreds of compound descriptions, toxic targets, mechanisms of toxicity, metabolism, uses, symptoms and treatment data fields have been added, re-written and/or expanded. Likewise hundreds of new references have been added or updated. Structures for nearly 250 compounds (that were previously missing their structures) have been included and a small number of existing structures were re-drawn or corrected to indicate proper stereochemistry.

Chemical nomenclature is particularly important in the chemical industry. Many commonly used industrial cleaners, solvents, pesticides, herbicides and drugs have dozens or even hundreds of alternate names, brand names or synonyms due to various trademark, patenting and/or marketing requirements. For the latest release of T3DB all of its synonyms and trade names were manually reviewed. Lengthy chemical names or meaningless catalog numbers were removed and only the most common synonyms were kept, enabling users to more easily browse alternate compound names. Brand names or synonyms appearing on more than one ToxCard were also identified and edited or removed as required.

A number of minor data field additions were also performed for the new release of T3DB. These include the addition of a ‘Biological Properties’ section covering a toxin's status (detected/quantified), origin (exogenous/endogenous), cellular locations, biofluid locations, tissue locations and pathways (SMPDB (23) or KEGG). Both normal and abnormal concentration data fields were also added so that users could better ascertain exposure levels. Likewise a number of new external links or database hyperlinks were added including those to the ChEMBL, OMIM and UniProt databases. Additionally MSDS data for a total of 2410 compounds have been added. These documents provide additional physico-chemical, handling, toxicity and treatment data that may not be present in a standard ToxCard.

### Interface improvements

**Figure 1.**
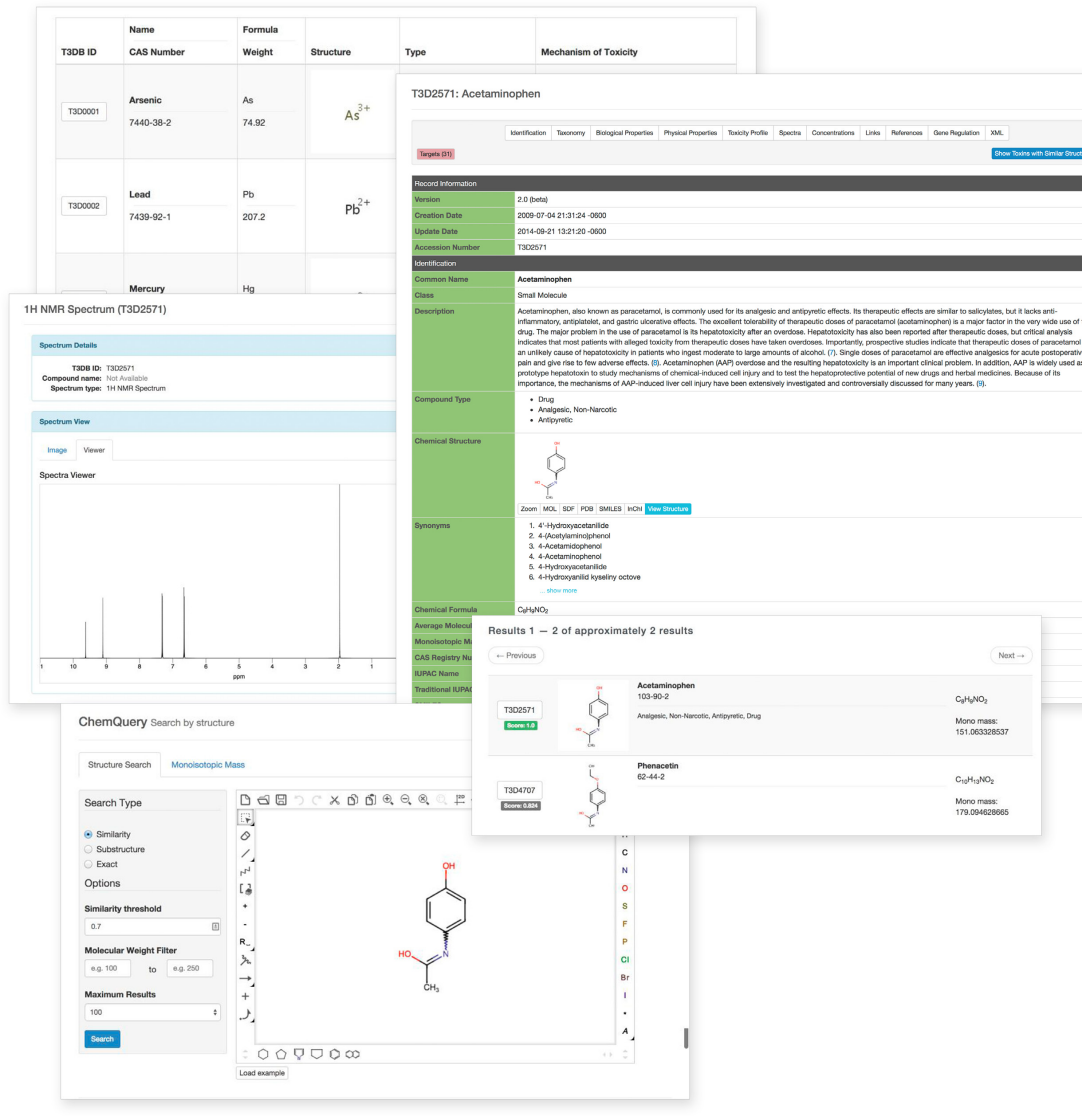
A screenshot montage of the Toxin, Toxin-Target Database (T3DB) showing several of T3DB's search and display tools describing Acetaminophen. Not all fields are shown. Clockwise from top left: Toxin Browse view; ToxCard for Acetaminophen; Chemical Structure Similarity Search for Acetaminophen; T3DB ChemQuery window; Spectral viewer for Acetamiophen.

With the significant data expansion and increasing number of data categories in T3DB we found it necessary to both revise and update the T3DB interface. This update included the addition of an improved and simplified browsing function for viewing toxic compound categories (reducing the number of categories from >400 to 17). It also included an upgraded Search menu to support MS, GC-MS and NMR spectral searches as well as the usual structure search (ChemQuery), sequence search, text search and advanced search functions (see Figure 1). To further facilitate browsing within each ToxCard we have added 12 ‘jump’ tabs that allow users to quickly jump to specific data categories without having to scroll through multiple pages or screens. These jump-tab categories include ‘Identification’, ‘Taxonomy’, ‘Biological Properties’, ‘Physical Properties’, ‘Toxicity Profile’, ‘Spectra’, ‘Concentrations’, Links’, ‘References’, ‘Gene Regulation’, ‘XML and ‘Targets’. We have also worked hard to improve the capabilities of T3DB's standard search tools. In particular, we have upgraded T3DB's text search engine to employ Elasticsearch (www.elasticsearch.org), an advanced and fully scalable enterprise search tool that is widely used in many computing science applications. This new search engine tolerates mis-spellings and provides suggestions or alternatives to facilitate further searching. With the new search engine it is now possible to search across toxin targets and other relevant search fields in T3DB. We have also upgraded T3DB's Advanced Search, thereby improving the granularity and coverage of the different data fields in T3DB. This should allow users to build much more complex queries through Advanced Search's simple interface thereby allowing them to search, retrieve or extract almost any kind of data type or combination of data within T3DB.

## CONCLUSION

T3DB is still a work in progress. As the fields of epidemiology, exposomics, metabolomics, toxicology, biochemistry and toxico-metabolomics evolve, so too will T3DB. This most recent release represents our best effort to bring the information in T3DB into better alignment with the needs of these diverse research communities. In particular, we believe the inclusion of a broader range of chronically toxic or non-acutely toxic compounds associated with diet, lifestyle and gut-microbe metabolism should make T3DB much more useful and appealing to researchers in epidemiology and exposomics. Similarly, the addition of biofluid concentration data along with NMR, MS and GC-MS spectra should make T3DB much more useful to researchers in exposomics, metabolomics and toxico-metabolomics. Likewise the inclusion of more target data with quantitative binding and/or activation information should make T3DB particularly appealing to toxicologists and biochemists.

With its unique emphasis on ‘common’ substances (i.e. those that can normally be detected with modern analytical methods), we believe the latest version of T3DB represents an important resource for exposure science research. As part of our re-branding of the T3DB as an exposome or exposure science database, we are also creating a mirror site (called the Toxic Exposome Database) and several alternate URLs (www.TEDB.ca and www.ExposomeDB.ca). Given T3DB's focus on depth as opposed to breadth, we are also hopeful that the rich content in this database will allow researchers, educators and members of the general public to gain a better appreciation of the toxic exposome and to uncover answers to many common questions about both the acute and the slow-acting poisons that are found in and around us.
